# Methamphetamine Inhibits Antigen Processing, Presentation, and Phagocytosis

**DOI:** 10.1371/journal.ppat.0040028

**Published:** 2008-02-15

**Authors:** Zsolt Tallóczy, Jose Martinez, Danielle Joset, Yonaton Ray, Attila Gácser, Sima Toussi, Noboru Mizushima, Josh Nosanchuk, Harris Goldstein, John Loike, David Sulzer, Laura Santambrogio

**Affiliations:** 1 Department of Neurology, Columbia University, New York, New York, United States of America; 2 Department of Pathology, Albert Einstein College of Medicine, Bronx, New York, United States of America; 3 Department of Physiology, Columbia University, New York, New York, United States of America; 4 Department of Medicine, Albert Einstein College of Medicine, Bronx, New York, United States of America; 5 Department of Microbiology and Immunology, Albert Einstein College of Medicine, Bronx, New York, United States of America; 6 Department of Bioregulation and Metabolism, The Tokyo Metropolitan Institute of Medical Science, Bunkyo-ku, Tokyo, Japan; 7 Department of Psychiatry, Columbia University, New York, New York, United States of America; 8 Department of Pharmacology, Columbia University, New York, New York, United States of America; University of Pennsylvania School of Medicine, United States of America

## Abstract

Methamphetamine (Meth) is abused by over 35 million people worldwide. Chronic Meth abuse may be particularly devastating in individuals who engage in unprotected sex with multiple partners because it is associated with a 2-fold higher risk for obtaining HIV and associated secondary infections. We report the first specific evidence that Meth at pharmacological concentrations exerts a direct immunosuppressive effect on dendritic cells and macrophages. As a weak base, Meth collapses the pH gradient across acidic organelles, including lysosomes and associated autophagic organelles. This in turn inhibits receptor-mediated phagocytosis of antibody-coated particles, MHC class II antigen processing by the endosomal–lysosomal pathway, and antigen presentation to splenic T cells by dendritic cells. More importantly Meth facilitates intracellular replication and inhibits intracellular killing of Candida albicans and *Cryptococcus neoformans,* two major AIDS-related pathogens. Meth exerts previously unreported direct immunosuppressive effects that contribute to increased risk of infection and exacerbate AIDS pathology.

## Author Summary

There is a new population of HIV+ men who are developing AIDS over months instead of years as typical. It has recently become popular among gay and bisexual men to consume very high levels of Meth. Unsafe sex together with Meth abuse has been suspected to lead to rapid disease progression. While studies show exacerbated AIDS symptoms and disease progression in HIV+ Meth abusers, the molecular mechanism is yet unknown. It was postulated, yet unproven, that the rapid disease progression might be due to a mutant “superstrain” of HIV that was extremely virulent. It was also assumed that the effects of the drug on behavior may lead to unsafe sex, although this would not explain the more rapid time course of the disease. We now demonstrate the first direct evidence that Meth is an immunosuppressive agent, and that the molecular mechanism of this immunosuppression is due to the collapse of acidic organelle pH in cells of the immune system, inhibiting the functions of antigen presentation, as well as phagocytosis. These effects compromise the immune response to opportunistic infections and HIV. These findings could have a major impact on public health, as there are over 35 million Meth abusers worldwide

## Introduction

Chronic methamphetamine (Meth) abuse has reached epidemic proportion throughout the United States, where a 2003 survey indicated that approximately 5% of the population over 12 years of age has tried Meth and the rate of treatment admissions for primary Meth abuse increased over 3-fold (The DASIS Report, http://www.oas.samhsa.gov/2k6/methTX/methTX.htm) [1,2]. In particular, among gay and bisexual men [3] Meth it is associated with high-risk sexual behavior, HIV viral infection, and a high incidence of AIDS [4]. Meth exacerbates AIDS pathology, including cognitive deficits [5,6], and is strongly suspected to inhibit normal immunological response to secondary infections such as hepatitis C, which is prevalent in those who smoke or take Meth intranasally [7]. It has recently been suggested that Meth could contribute to a particularly rapid progression of AIDS in individuals exposed to a strain of HIV that is resistant to drug treatment [8,9]. Indeed, animal studies clearly demonstrate that Meth suppresses both innate and adaptive immunity [10,11], enhances cytokine production in combination with HIV TAT protein [12], and alters gene expression in cells of the immune system [13]. However, the molecular basis for Meth's immune suppression is unknown. Here we have examined the relationship between Meth and the impairment of specific immune cell functions.

In the clinical setting, Meth abusing individuals who present with opportunistic infections possess high blood and tissue levels of the drug. Meth is generally self-administered in this population in binges of 3–4 grams ingested over a six day interval [14], with an initial ingestion of ∼0.5 gram (Judith Rabkin, Columbia University, personal communication) and a total mean level of 2.2 grams ingested during the first day [15]. Such high levels of administration result in a blood concentration of ∼10–50 μM Meth, and levels in the hundreds of micromolar range in organs including brain and the spleen [16] (see Results).

Pathogens are processed and displayed by antigen presenting cells (APCs) for T cell recognition. Antigen presentation in tissue resident macrophages, as well as dendritic cells, involves fluid phase or receptor-mediated endocytosis followed by fusion of the phagosome with specialized lysosomes known as MHC class II compartments (MIIC) [17]. The foreign antigens are partially degraded by lysosomal hydrolases and the resulting peptides are loaded on MHC class II molecules and transported to the cell membrane to be presented to T cells. Both pathogen transport to the MIIC and its degradation into immunogenic peptides are functions that require an acidic endosomal pH. Endogenous antigens and viruses can be delivered to the MIIC upon autophagosome fusion [18]. Autophagy also mediates single-stranded RNA virus detection and consequent interferon-α release in plasmacytoid dendritic cells activating anti-viral cellular defense mechanisms in uninfected cells [19]. Further evidence shows the direct role of autophagosomal-lysosomal degradation in elimination of intracellular HSV-1 viral particles [20] and that the HSV-1 has alternative methods to counteract cellular autophagy [21].

In this study, we have identified a novel molecular mechanism that explains the immunosuppressive effects of Meth via the alkalization of acidic organelles in dendritic cells and macrophages that are critical for the immunological function of these APCs.

## Results

### Estimates of Pharmacological Meth Levels in Humans

To model effects of Meth on the immune system, we estimated Meth levels in drug abusers. Meth is self-administered intravenously, by nasal inhalation, anally, and orally, in typical doses of 250–500 mg by occasional users to levels as high as 1 g by chronic abusers (personal communication, Perry N. Halkitis, New York University). Meth blood levels measured in individuals detained by police in California were 2.0 μM on average but as high as 11.1 μM [22]. Controlled studies indicate that a single 260 mg dose reaches a level of 7.5 μM [22]. Thus, a single dose of 260 mg – 1 g would be expected to produce 7.5 – 28.8 μM blood Meth levels.

The abusers however tend to self-administer METH in binges, and as the drug exhibits a half-life of 11.4 - 12 h [23,24], this leads to higher levels. Recently published studies modeling binge pattern of use in individuals show that after the fourth administration of 260 mg during a single day produces maximum blood levels of 17 μM, reaching 20 μM on the second day of such a binge [22]. Thus, binge doses of 260 mg – 1 g would produce 17 - 80 μM blood Meth levels. The estimates appear consistent with blood levels detected after fatalities [25–27], ranging as high as 84 μM for an individual for whom Meth intoxication was determined as the cause of death ∼16 hours after ingestion.

It is also important to estimate how Meth is distributed from blood to other tissues involved in immune response, particularly the spleen, which houses high numbers of dendritic cells. Tissue-to-serum Meth ratios in rat are: brain, 9.7; kidney, 35.3; spleen, 14.3 [16]. Thus, relevant levels in spleen, the organ critical for immune response, after administration of 250 mg – 1 g as a single dose is 100 – 400 μM, and during binges between 240 – 1144 μM.

### Meth Induces Rapid Alkalization of Acidic Organelles and Changes in Endosomal Morphology

**Figure 1:**
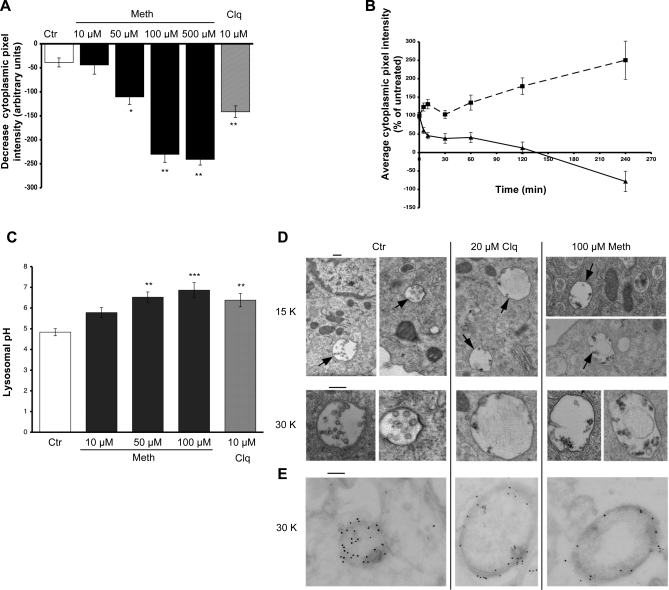
Meth Induces Rapid Alkalization of Acidic Organelles, and Changes in Endosomal Structure in Dendritic Cells (A) Morphometric analysis of the decrease in acridine orange staining after 10 min treatment. Similar results were obtained in three independent experiments. (Statistics *n* = 25–35, mean ± SEM, **p < 0.001, ***p < 0.0001, two-tailed ANOVA.) (B) Progressive quenching of cytosolic acridine orange staining over 4 h in the absence () or presence (▴) of 50 μM Meth. *p* < 0.001 for all time points versus control (*n* = 25–35, mean ± SEM, Student's two-tailed t-Test). (C) Acidic organelle pH in cells exposed to Meth for 10 min measured by ratiometric measurement. (Statistics *n* = 30–90 cells, mean ± SEM, **p < 0.001, ***p < 0.0001, two-tailed ANOVA.) (D) Representative electron micrographs showing multivesicular endosomal compartments (arrows) in control, Clq- and Meth-treated cells at 4 h. Scale bar, 200 nm. (E) Ultra-thin cryosections of control, Clq- and Meth-treated dendritic cells as in (D), immunolabeled for LAMP-1 (15 nm gold particles) and MHC class II (10 nm gold particles). Scale bar, 200 nm.

Dendritic cell MIIC processing organelles are characterized by acidic pH [28], a limiting membrane enclosing internal vesicles or lamellae [17], the presence of proteases [29], internal expression of LAMP [30] and MHC II proteins [31]. The morphological and functional integrity of the MIIC depends on the maintenance of an acidic pH [32] due to the action of the V-ATPase, which may also regulate transport from early to late endosomes and lysosomes [33]. Meth and its metabolite, amphetamine, are membranophilic weak bases that collapse intracellular organelle pH gradients in neurons [34,35]. We therefore tested whether Meth collapsed intracellular organelle pH gradients in dendritic cells by monitoring quenching of acridine orange, a weak base vital dye that accumulates in acidic organelles including endosomes and lysosomes [35]. We found that Meth concentrations of 50 μM or higher rapidly collapsed acidic organelle pH gradients in dendritic cells (Figure 1A–1B). LysoSensor Yellow/Blue ratiometry is a generally accepted method to measure organellar pH in live cells, and was used to measure average pH in acidic organelles using LysoSensor Yellow/Blue fluorescent dye. After 10 min treatment with Meth (50 or 100 μM) or chloroquine (10 μM) acidic organellar pH was elevated (pH 6.5, 6.9 and 6.4 respectively) significantly above the levels in untreated control cells (pH 4.8) (Figure 1C). As predicted with alkalizing agents, Meth also disrupted MIIC structure [36], producing large organelles (> 1 μm diameter) devoid of internal vesicles (right panels) with LAMP-1 and MHC II staining confined to the limiting membrane (Figure 1D and 1E). Chloroquine (Clq) [10 or 20 μM], another weak base was used as a control showed similar effects to Meth as described above (Figure 1A–1E). At the used concentrations (20 and 100 μM) Meth did not affect cell viability as determined by flow cytometric analysis (Figure S1).

### Meth Inhibits Endosomal Antigen Processing and MHC II Restricted Antigen Presentation

**Figure 2:**
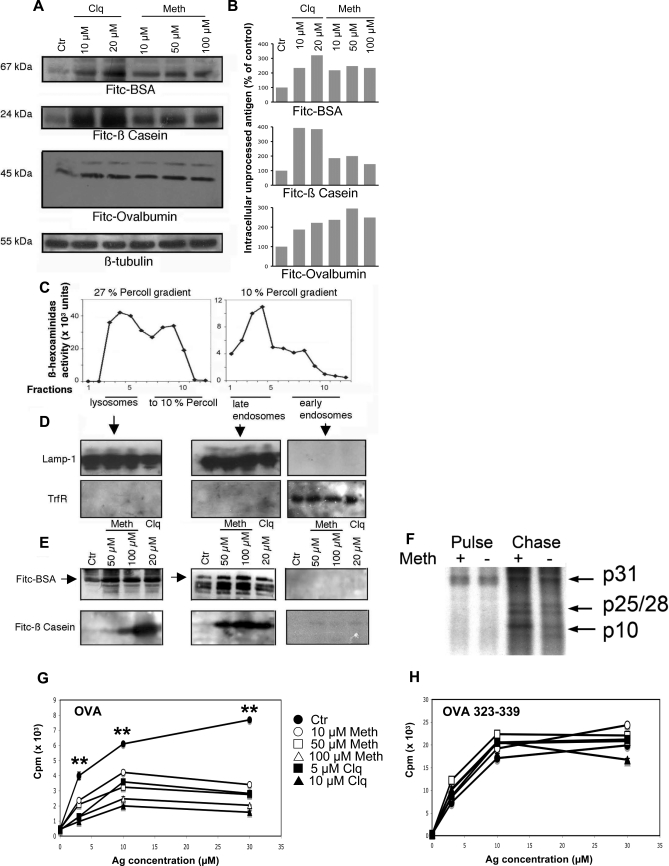
Meth Inhibits Endosomal Antigen Processing and MHC II Restricted Antigen Presentation (A) Western blots of bone marrow derived dendritic cells which phagocytosed FITC-labeled BSA, casein or OVA overnight, and exposed to Clq or Meth. Unprocessed antigens are quantified using anti-FITC mAb. (B) Densitometric analysis of western blots for the amount of unprocessed intracellular antigen. Representative of two to three independent experiments. (C) β-hexosaminidase activity measured in sub-cellular fractions to identify lysosomes and late endosomes. (D) Western blot using LAMP-1 and TrfR mAb on lysosomal, as well as early and late endosomal fractions. (E) Western blot analysis of the above pulled fractions from untreated, Clq- or Meth-treated dendritic cells using anti-Fitc mAb. (F) Immunoprecipitation of invariant chain in pulse-chase experiments. Full length invariant chain (p31) and degradation products (p25/28 and p/10) are indicated. (G) T cell proliferative response to bone marrow derived dendritic cells cultured with OVA protein (0, 3, 10, and 30 μM) in the absence (Ctr •) or presence of Meth (10 ○, 50 □, 100 μM ▵) or Clq (5 ▪ , 10 μM ▴) for 6 h, and cultured with T cells for 3 d. [^3^H]-Thymidine incorporation was used to assay T cell proliferation. (Statistics *n* = 4, mean ± SEM, **p < 0.001, two-tailed ANOVA.) (H) T cell proliferative response to bone marrow derived dendritic cells cultured with OVA peptide 323–339 (0, 3, 10, and 30 μM) in the absence (Ctr •) or presence of Meth (10 ○, 50 □, 100 μM ▵) or Clq (5 ▪ , 10 μM ▴) for 6 h, and cultured with T cells for 3 d.

To investigate whether Meth-induced endosomal alkalization blocks antigen processing by impairing dendritic cell lysosomal proteolytic degradation of foreign proteins, we exposed cells to the fluorescently labeled MHC II antigens [bovine serum albumin (BSA), casein, and ovalbumin (OVA)], and measured the degradation of each protein by western blot. In untreated cells, each antigen was proteolytically degraded, while antigen degradation was blocked in Meth or Clq treated cells (Figure 2A and 2B). Meth (10, 50, 100 μM) and Clq (10, 20 μM) effectively inhibited processing of antigens previously taken up by dendritic cells (Figure 2A). Control experiments showed that Meth did not block endocytosis of 2 μm fluorescent dextran beads by dendritic cells (Figure S2), indicating that the drug does not inhibit nonspecific phagocytosis [37] .

To determine the stage at which Meth compromised post-endocytic proteolytic antigen processing, we prepared fractions of early and late endosomes and lysosomes from dendritic cells exposed to BSA and casein antigens. Each fraction was examined for β−hexosaminidase to identify lysosomes and late endosomes, the transferrin receptor (TrfR) to identify early endosomes, and LAMP-1 to identify late endosomes and lysosomes (Figure 2C and 2D). No detectable antigen remained in early endosomes under any of these conditions. There were, however, much higher levels of BSA and casein in lysosomes and late endosomes of Meth- and Clq-treated cells than in controls. In particular, casein was completely degraded in untreated cells but relatively unprocessed in dendritic cells treated with Meth or Clq (Figure 2E). Thus, Meth inhibited antigen proteolysis within late endosomal/lysosomal compartments. Similarly, processing of invariant chain was compromized after Meth treatment as indicated by increased levels of p25/28 and p10 fragments during the chase time-point (Figure 2F).

MHC II antigen presentation stimulates T cell proliferation, providing a means to measure effects of Meth on antigen presentation. We prepared cultures of immature bone marrow-derived dendritic cells and splenic purified T cells, both from OTII transgenic mice [38]. Using OVA as an antigen, we assayed cellular proliferation of T cells by radiolabeled thymidine uptake and incorporation into DNA. At all levels tested, Meth decreased the T cell proliferative response to the intact antigen (Figure 2G) but not the pre-processed OVA-323–339 peptide (Figure 2H).

### Autophagosome Accumulation Is Induced by Meth

**Figure 3:**
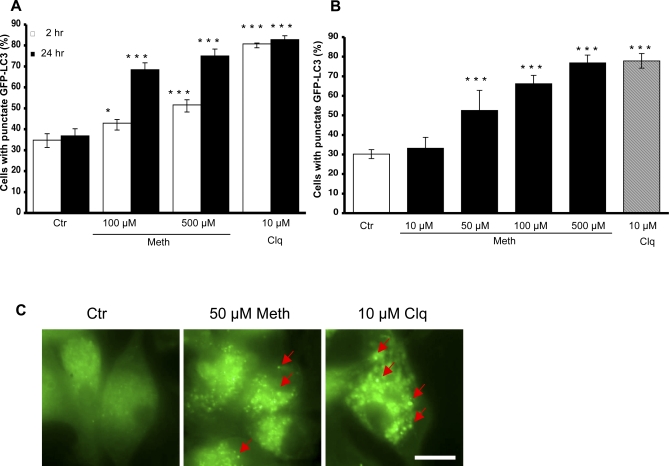
Meth Induces Autophagosome Accumulation in Dendritic Cells (A) Accumulation of GFP-LC3 labeled autophagosomes in Meth- or Clq-treated dendritic cells at 2 h and 24 h. Fisher's exact test P values versus control (*n* =300–400 cells, mean ± SEM, *p < 0.05, ***p < 0.0001). (B) GFP-LC3 labeled autophagosome formation in different concentrations of Meth after 24 h treatment. Similar results were obtained in three independent experiments. Fisher's exact test versus control (*n* = 300–400 cells, mean ± SEM, ***p < 0.0001). (C) Representative fluorescence micrographs of GFP-LC3 decorated autophagosomes in cells treated with 50 μM Meth or 10 μM Clq for 24 h. Arrow denotes examples of autophagosomes. Scale bar, 10 μm.

In addition to blocking lysosomal antigen degradation, Meth could disrupt processing and presentation of antigens by inhibiting autophagosome formation, thereby halting antigen delivery to MIIC. To test this possibility, we prepared dendritic cells from a transgenic mouse expressing a GFP-fused autophagosome-associated protein LC3 (GFP-LC3) which has been used as an in vivo autophagosomal marker [39]. Consistent with previous reports in neurons [40], 50–500 μM Meth induced autophagosome accumulation in dendritic cells (Figure 3A–3C). Our data show that Meth does not block autophagosome formation, but rather impairs lysosomal-autophagosomal degradation, resulting in the accumulation of autophagosomes, and impaired intracellular antigen proteolysis. Together with the evidence from endosomal/lysosomal fractions (Figure 2C–2E), these results strongly suggest that Meth inhibits degradative antigen processing by disrupting pH gradients.

### Meth Alkalizes Acidic Organelles and Induces Autophagosome Formation in Macrophages

As with dendritic cells, we also found that Meth and Clq effectively collapsed the intracellular pH gradients within macrophages (Figure S3A–S3C) and blocked autophagosome degradation, resulting in the accumulation of GFP-LC3 labeled autophagosomes (Figure S4A–S4D). Similar results were obtained with the fluorescent dye monodansylcadaverine, a lipophilic weak base that accumulates in lysosomes and autophagosomes (Figure S4E).

### Meth and Clq Inhibit Phagocytosis in Macrophages

**Figure 4:**
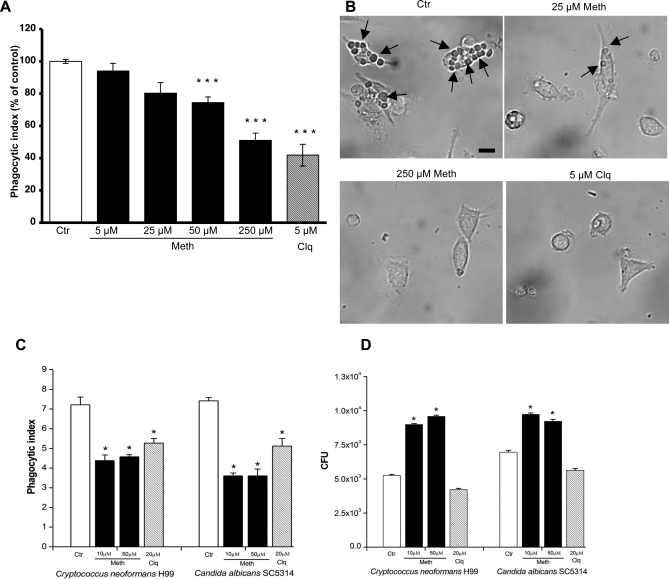
Meth and Clq Inhibit Phagocytosis in Mouse Peritoneal Macrophages (A) Cells were incubated in indicated concentrations of Meth or Clq for 2 h, and then IgG coated erythrocytes were added. Extracellular uningested EIgG were lysed and removed. Phagocytic index (PI) was quantified and inhibition is indicated as percent of control. Data were collected from four to seven experiments (*n* = 300 cells, mean ± SEM, ***p < 0.0001, two-tailed ANOVA). (B) Images of macrophage cells impaired in the phagocytosis of opsinized sheep erythrocytes after 2 h of Meth or Clq treatment. Arrows denote phagocytosed erythrocytes. Scale bar, 10 μm. (C, D) J774.16 cells were exposed to PBS, chloroquine, or methamphetamine for 2 h followed by incubation with Cn or Ca. The phagocytic indices (ratio of intracellular yeast to the number of macrophages counted) were determined after 1 h or 30 min for Cn and Ca, respectively (C). (Statistics *n* = 300, mean ± SEM, *p < 0.05, two-tailed ANOVA.) CFU determinations were preformed after 24 h incubations (D). The values are the mean and standard deviation from determinations made in triplicate wells (*n* = 300 cells, mean ± SEM, *p < 0.05, two-tailed ANOVA.). Experiments were repeated three times with similar results.

Since Meth users can also present with bacterial infections [41], we examined whether Meth disrupts macrophage phagocytosis, a primary mechanism for clearance of these extracellular pathogens. Murine peritoneal-derived macrophages were incubated in the presence or absence of Meth. After 2 h, IgG-antibody coated erythrocytes [E(IgG)] were added to each of the conditions and the number of ingested erythrocytes counted. We found that Meth (50 and 250 μM) inhibited E(IgG) phagocytosis (Figure 4A and 4B) by 20 and 45%, respectively. Meth did not inhibit the receptor-independent endocytosis of Lucifer Yellow (Table S1). Similar effects were observed with Clq (Figure 4A and 4B) These results demonstrate that Meth inhibits Fcγ-mediated phagocytosis in macrophages and are consistent with the observations that the macrophage Fcγ receptors are continuously recycled between phagosomes and the plasma membrane [42], a process that requires appropriate acidification of secretory vesicles and tubules for trafficking [43]. These results demonstrate that Meth inhibits Fcγ-mediated phagocytosis in macrophages.

### Meth and Clq Inhibit Macrophage Phagocytosis and Intracellular Replication of Candida albicans and Cryptococcus neoformans

As a next step we analyzed the effects of Meth on Candida albicans (Ca) and Cryptococcus neoformans (Cn) phagocytosis and killing by murine macrophages since these two organisms are the most commonly isolated fungi in individuals infected with HIV [44]. Clq and Meth (10 and 50 μM) inhibited phagocytosis of Ca and Cn by macrophages by 40% (Figure 4C). Moreover, Meth enhanced the proliferation of fungi within macrophages (Figure 4D), indicating that intracellular replication of both yeast was facilitated by Meth. In contrast, Clq had no significant, or slightly reducing, effect on Ca (*p*= 0.056) and Cn (*p* = 0.060) CFU numbers in macrophages. Control experiments showed that in the absence of macrophages, Ca and Cn proliferation was unaffected by the addition of Clq or Meth to the BHI medium (data not shown).

### Meth-Treated HIV-Infected Macrophages Exhibit Increased p24 Secretion

To further examine why chronic Meth abuse has been recently associated with the rapid development of immune deficiency among gay and bisexual men [3], we studied the effect of Meth on HIV proliferation in macrophages from HIV-transgenic mice. These JR-CSF/huCycT1 double transgenic mice express HIV-1 JR-CSF, which is a full length R5 HIV-1 provirus regulated by the endogenous HIV-1 LTR, as well as the human cyclin T1 controlled by a murine CD4 expression cassette [45]. These mice have constitutive HIV production in CD4 T lymphocytes and monocytes. GM-CSF differentiated bone marrow cells were either left untreated or treated with a range of Meth levels (10, 50, 150 μM) or NH_4_Cl (10 mM), another weak base, for 7 days. Cell supernatants were then collected and p24, a secreted HIV-specific protein, was quantified by Elisa. Cells from JR-CSF/huCyc T1 mice treated with Meth for 7 or 9 days exhibited a 30–60% increase (Figure S5A) of p24 antigen production. Clq does not provide a positive control, since its well-established inhibition of HIV production is probably due to inhibition of viral capsid protein glycosylation in the Golgi [46]. Thus, in this experiment NH_4_Cl was used as positive control (Figure S5A).

To determine whether HIV replication was affected by Meth in vivo, we studied the effect of Meth on HIV virus proliferation in the JR-CSF/huCycT1 double transgenic mice [45]. Mice were treated with increasing concentration of Meth over a 7 day-period. One group of animals received 5 mg/kg of Meth at day 0, 2 and 4 and was sacrificed at day 6 (low Meth). Another group received 6 mg/kg at day 0; 7 mg/kg at day 2 and 7.5 mg/kg at day 4 (high Meth) and was also sacrificed at day 6. Copy number of HIV-1 RNA was quantified in the serum of each mouse by RT-PCR using primers spanning the highly conserved region of the HIV-1 gag gene. No statistically significant differences were observed between the untreated and the Meth-treated mice (Figure S5B). These data suggest that Meth treated HIV-1 transgenic mice do not exhibit an increase in HIV viral load.

## Discussion

We find that the widely abused addictive psychostimulant, Meth, at pharmacologically relevant levels acts as an immunosuppressive agent, due to its inhibition of endosomal acidification. These actions result in Meth's inhibition of antigen presentation and phagocytosis. Maintenance of low endosomal and lysosomal pH serves many functions, including regulation of protein degradation, pathogen inactivation, and regulation of the amount of several surface receptors. All of these functions require active transport via the endocytic pathway and fusion with lysosomal compartments.

First, via alkalization, METH-inhibited lysosomal-autophagosomal degradative function for both exogenously and endogenously internalized antigens resulting in accumulation of proteins entering the endocytic pathway through phagocytosis, as well as autophagic vacuoles. Chaperone-mediated autophagy and macroautophagy in lysosomes have been described as major pathways for endogenous antigen processing in MHC class II compartments [18,47,48] and as a means to directly degrade intracellular virus particles [20,21]. Thus, through its alkalizing effect, Meth blocks normal antigen processing and presentation. Progression of internalized antigens along the endocytic pathway rely on the progressive maturation of early endosomes into late endosomes and ultimately to lysosomes. Maintenance of an acidic internal pH and a pH gradient in these compartments is important for the cargo progression. Endosomal acidification is accomplished by H^+^ transport across the endosomal limiting membrane by the proton pump vacuolar ATPase (V-ATPase). The recent discovery that V-ATPase interacts with components of the endocytic transport machinery indicates that V-ATPase is also a pH sensor that regulates early to late endosomal transport [33]. This would explain why endosomal alkalization by Meth not only disrupts antigen processing but phagocytosis and cargo progression along the endosomal pathway.

Second, endosomal alkalization by Meth inhibited *Ca* and *Cn* phagocytosis and killing by macrophages. These effects are expected to be particularly devastating in AIDS-related disorders, since *Ca* and *Cn* are the two most commonly isolated fungi from sterile body fluids obtained from HIV infected individuals [44]. Also, these results could explain recent reports of rapid insurgence of AIDS in Meth-addicted individuals soon after infection with HIV. Thus, Meth also blocks pathogen killing by macrophages. Exposure to Clq and other weak bases was already been shown to inhibit growth of Cn in macrophages [49,50] even though Clq has no direct toxicity to Cn [50]. Not all basic compounds are equivalent in their fungicidal activity; for example, ammonium chloride also enhances the anti-cryptococcal activity of macrophages, yet the potency of ammonium chloride is less than that of Clq, which may be a result of the ability of ammonium chloride to inhibit phagolysosomal fusion [51]. Hence, Meth apparently has complex effects on macrophages that result in an intracellular milieu that enables the replication of the examined pathogenic fungi.

Third, even though an increase in p24 secretion has been observed in Meth-treated dendritic cells and macrophages, a direct effect of Meth on HIV viral load could not be demonstrated. It is likely that viral proteins enter late endosomal and lysosomal compartments independently of viral assembly, and due to inhibition of processing, more viral proteins are secreted in the extracellular milieu. Within the infected macrophages, HIV has been previously shown to assemble in compartments with characteristics of multivesicular late endosomes (CD63, Lamp-1, CD81 and CD82 positive) [52]. It now appears that the HIV particles present in multivesicular endosomes are the results of endocytosis, and the site of viral assembly is at the invagination of the plasma membrane particularly enriched in tetraspannin proteins [53]. These results would explain why, by compromising endosomal pH, an increase in HIV viral load has not been observed.

In conclusion, the immunosuppressive effects of Meth are consistent with reports that Meth-treated mice demonstrate decreased immunity [10,11]. To be noted is that even though there is a linear concentration-dependent response between Meth concentration and pH disruption such an effect is less evident at the biological level. In all assays (antigen processing, Ca or Cn killing and p24 production) different Meth dosages behave very similarly. Likely a small disruption in the endosomal pH is sufficient to alter the microenvironment and endosomal-related functions. This is consistent with the effects of low concentrations of Meth tested on lysosomal pH in Figure 1C, with alkalinization of > 1 pH unit, which would effectively inhibit lysosomal protease activity.

The collapse of endosomal pH by Meth and the resulting decrease of normal immune response provide an explanation for the compromised immunity and exacerbate infections occurring in Meth abusers. In fact, Meth is strongly suspected to more dramatically inhibit normal immune responses than other drugs of abuse since Meth users often present with skin lesion and “Meth mouth”, a devastating periodontal disease (http://www.drugfree.org/) [54] and [1,2]. A similar immunosuppressive activity has also been shown for chloroquine, also a well known inhibitor of endosomal acidification [36].

In particular, Meth immunosuppression may underlie the mechanism of the recently reported extremely rapid development of immune deficiency, with devastating effects in AIDS-related disorders in Meth abusers that had contracted HIV. In particular, there is evidence suggesting the presence of a new population of HIV^+^ positive men who are developing AIDS over months rather than over 10 or more years as is typical. The most widely reported individual was documented by Dr. Martin Markowitz of the Aaron Diamond AIDS Research Center in New York in 2004. In this individual, a gay man who was also a Methabuser tested HIV negative in May, 2003, then likely contracted HIV during unprotected sex in mid-October 2004, displayed acute retroviral syndrome in November, 2004 and 3-drug-class-resistant HIV-1 (3DCR HIV) with apparently rapid progression to AIDS by December, 2004 (CDC, MMWR July 28, 2006/55(29);793–796). Meth self-administration by HIV+ individuals during the acquisition of sexually transmitted pathogens appears likely to interfere with immunological resistance and lead to AIDS progression.

## Materials and Methods

Additional detailed methods used in this paper can be found in Protocol S1.

### Primary cell culture.

Mouse femur hematopoietic stem cells from bone marrow were harvested from the hind legs of 8 to 12-week old male wild-type C57BL/6J (The Jackson Laboratory, Bar Harbor, Maine) or GFP-LC3 transgenic mice [39], and plated at 2 × 10^6^ cells/ml density in DMEM supplemented with 10% FBS, 1x non-essential amino acids (Gibco, Carlsbad, California), 2 mM L-glutamine, 1 mM sodium pyruvate and 20 mM HEPES. For differentiation to dendritic cells or macrophages, 10 ng/ml of recombinant mouse GM-CSF (Biosource, Carlsbad, California) or 10 ng/ml of recombinant mouse M-CSF (R&D Systems, Minneapolis, MN) was added to media, respectively. Cells were fed every 2 days with fresh DMEM containing the appropriate macrophage colony stimulating factor. Cells were trypsinized after 8 days and, unless otherwise noted, plated at 4 × 10^5^ cells/cm^2^ density to be used for experiments the following day.

Resident mouse peritoneal macrophages were isolated from 8 to 12 week-old female wild-type C57BL/6J as described [55] and plated on 12 mm-diameter glass coverslips in 24-well tissue culture plates at 3 × 10^5^ cells/well density in RPMI media supplemented with 10% FBS with streptomycin and penicillin. Non-adherent cells were removed by washing 2 h after plating. The remaining adherent cells were over 95% macrophages as assessed by esterase staining [55]. Cells were incubated overnight prior to experiments.

### Morphometric analysis of acridine orange staining.

Cells were stained with 10 mM acridine orange in phenol red-free media for 1 h. Images of stained cells were acquired using fluorescence microscopy as described above. Images of 10 fields with 5 to 10 cells for each image were taken using multiple stage positions in Multidimensional Acquisition mode under conditions of no photobleaching (ND2 filter, 1000 msec exposure) that enabled the acquisition of multiple images of the same cells. Stage position for each image was stored so images of the same fields and cells could be taken before and after treatment. Phenol red-free media containing 10 mM acridine orange in the presence or absence of Meth or Clq was added to the cells. At the end of the incubation time, images of treated cells were taken and used for morphometric analysis. Change in acridine orange intensity was measured as a change in average pixel intensity in the cytoplasmic area using MetaMorph Version 6.1r6 image analysis software (Molecular Devices, Sunnyvale, CA). Background mean pixel intensity was measured in nuclear area and subtracted.

### Ratiometric measurement of acidic organelle pH.

Dendritic cells were stained with 5 μM LysoSensor Yellow/Blue (Invitrogen, Carlsbad, CA) for 5 min before Meth (10, 50, 100 μM) or Clq (10μM) was added for an additional 10 min incubation followed by washing with phosphate-buffered saline (PBS) (pH 7.4). Fluorescent images were taken of the same cells using Olympus IX81 microscope with Photometrics CoolSNAP HQ cooled camera, MetaMorph Version 6.1r6 imaging software (Molecular Devices, Sunnyvale, CA), Olympus PlanApo 40x/1.4 Oil objective, equipped with fluorescent yellow customized Chroma (D350/50 excitation, 400DCLP dichroic splitter and D535/40m emission) (Chroma technology Corp., Rockingham, VT) and blue Chroma 31000v2 (D350/50 excitation, 400DCLP dichroic splitter and D460/50m emission) filter sets. Average pixel intensity was measured in the cytoplasm of the cells excluding the nucleus using MetaMorph software, and the ratio of yellow to blue intensity was compared to a pH calibration curve to determine pH values. For the calibration curve, cells were stained with 5 μM LysoSensor Yellow/Blue for 20 min, washed with PBS and incubated in buffer of known pH (4.0 to 7.4) containing 10 μM monensin and 10 μM nigericin [56] before images were taken and processed as above.

### Ultrastructural analysis and immunogold labeling.

Dendritic cells were derived and incubated for 4 h with or without Clq (20 μM) or Meth (100 μM). Cells were fixed in 2% paraformaldehyde and 2.5% glutaraldehyde in 0.1 M sodium cacodylate buffer, postfixed with 1% osmium tetroxide followed by 1% uranyl acetate, dehydrated through a graded series of ethanol and embedded in LX112 resin (LADD Research Industries, Williston, VT). Ultrathin (80 nm) sections were cut on a Reichert Ultracut UCT, stained with uranyl acetate followed by lead citrate and viewed on a JEOL 1200EX transmission electron microscope at 80 kV.

For immunogold labeling, cells treated as described above were fixed in 2% paraformaldehyde and 4% polyvinylpyrolodone in phosphate buffer 0.2 M (pH 7.4) at 4 °C. Fixed cells were processed for ultrathin cryosectioning as previously described [14]. Immunogold labeling was performed using LAMP-1 antibody (clone 1D4B, BD Pharmingen, San Diego, CA) followed by anti rat Ig-G coupled with 10 nm gold particles and biotinylated anti-MHC II (clone M5/114.15.2, BD Pharmingen, San Diego, CA) followed by streptavidin gold (15 nm). Contrast was obtained with a mixture of 2% methylcellulose (Sigma, St. Louis, MO) and 0.4% uranyl acetate pH 4 (EMS, Hatfield, PA).

### Antigen processing.

FITC-coupled BSA, ovalbumin or casein were fed to immature bone marrow-derived dendritic cells (between 1 to 3 × 10^7^ cells for each condition) at a concentration of 100 μg/ml. Cells were untreated or treated with Meth or Clq. After overnight incubation, cells were washed twice in PBS and lysed in 150 mM NaCl, 50 mM Tris-HCl and 1% NP40 supplemented with protease inhibitor cocktail. Post-nuclear supernatants were normalized for protein content and 80 μg of total protein was run on SDS-PAGE gel. Membrane blots were probed with the anti FITC mAb or β-tubulin mAb (Sigma, St. Louis, MO).

### Sub-cellular fractionation.

The immature bone marrow-derived dendritic cell line JAWS (ATCC) was used for sub-cellular fractionation. One hundred million cells were used for each experimental condition. Cells (4 × 10^6^ cells/ml) were incubated overnight with 80 μg/ml of FITC-labeled BSA or casein in presence or absence of Clq (20 μM) or Meth (50–100 μM). Cells were then lysed in 250 mM sucrose, 1 mM EDTA pH 7.4. Early and late endosomes and lysosomes were prepared over consecutive Percoll gradients (27% and 10%) from cells treated as reported above [57]. Each fraction was tested for β−hexosaminidase to locate lysosomes and late endosomes. The late endocytic marker Lamp-1 (clone 1D4B, BD Pharmingen, San Diego, CA) and the early endosomes/plasma membrane marker transferrin receptor (TrfR) (clone M-A712, BD Pharmingen, San Diego, CA) were also used to assess the purity of the endosomal preparations. Pulled fractions 3–6 from the 27% Percoll gradient (lysosomes), 2–5 from the 10% Percoll gradient (late endosomes) and 7–10 from the 10% Percoll gradient (early endosomes) were run on SDS-PAGE and blotted membranes analyzed for FITC-labeled antigens as reported above.

### Pulse chase and immunoprecipitation.

GM-CSF differentiated bone marrow dendritic cells were cultured in methionine- and cysteine-free medium complete DMEM media containing 5% dialyzed serum for 1 h. Cells were then labeled with 0.2 mCi/ml [^35^S]-methionine (Perkin Elmer, Waltham, MA) for 30 minutes (pulse). Cells were then washed three times and incubated in complete DMEM media supplemented with 10X cold methionine for 4 hours (chase) in the presence or absence of 50 μM Meth. Cells were subsequently lysed in 1% NP40, 150 mM NaCl, 50 mM Tris containing a cocktail of protease inhibitors (Complete Mini, Roche Diagnostics, Indianapolis, IN) for 30 min on ice, spun at 14000 rpm for 30 minutes to remove cell nuclei and debris. The amount of incorporated radioactivity in each sample was determined by precipitating 10 μl of the post-nuclear supernatants with 10% trichloroacetic acid (TCA). Equivalent amounts of radioactive lysates were pre-cleared with rat serum adsorbed to Prot G beads followed by Protein G beads alone, for 2 hours at 4°C. Immunoprecipitation was performed using 10 μg of anti CD74 (clone In-1, Pharmingen, San Diego, CA) bound to Protein G beads. The beads were washed 3 times with lysis buffer and eluted with sample buffer. The elute was boiled and resolved by SDS-PAGE. The gel was subsequently dried and exposed in autoradiography.

### Antigen presentation.

Bone marrow-derived dendritic cells from OT II transgenic mice (Jackson Laboratory, Bar Harbor, MN) were grown in 10 ng/ml of mouse GM-CSF for 10–12 days. Splenic T cells were purified using the pan-T cell isolation kit (Miltenyi Biotec, Auburn, CA) according to the manufacturer's suggestions. One hundred thousand dendritic cells were cultured with 4 × 10^5^ T cells in the presence or absence of 0, 3, 10, and 30 μM OVA protein for 3 days at 37°C. In some experiments Meth or Clq was added on the first day of culture until the end of the proliferative response. In other experiments, dendritic cells were pretreated with Meth or Clq for 4 h at 37°C, washed and fixed in 1% paraformaldehyde before adding OVA 323–339 peptide and the T cells. In all experiments, [^3^H]-thymidine (1 μCi/well) was added during the last 18 h of incubation to assay T cell proliferation. Plates were harvested and the DNA [^3^H]-thymidine incorporation was monitored using a Wallac liquid scintillation counter (Perkin Elmer, Waltham, MA).

### Autophagy assay in GFP-LC3 dendritic cells and macrophages.

Bone marrow-derived dendritic cells and macrophages from GFP-LC3 animals were plated at 4 × 10^5^ cells/cm^2^ density in glass bottom dishes and incubated overnight. Meth or Clq was added to the media, and the cells incubated for 2 h and 24 h before fluorescence microscopy using an Olympus IX81 microscope with Photometrics CoolSNAP HQ cooled camera, MetaMorph Version 6.1r6 imaging software (Molecular Devices, Sunnyvale, CA), TC-324B Automatic Temperature Controller (Warner Instrument Corporation, Hamden, CT), Olympus PlanApo 60x/1.4 Oil objective, and a Chroma FITC 41001 (HQ480/40x excitation, Q505LP dichroic splitter and HQ535/50m emission) (Olympus, Center Valley, PA) to determine GFP-LC3 puncta formation in the cells. At least 100 cell profiles per dish were assayed in triplicate for GFP-LC3 puncta.

### Macrophage phagocytosis assay and colony forming unit (CFU) determination.

Mouse peritoneal macrophages at 3.5 × 10^5^ cells/cm^2^ density on cover slips were incubated at 37°C with 20 μl of IgG opsinized sheep erythrocytes in 520 μl volume for 90 min. Uningested erythrocytes were lysed by sequential washing with PBS, water, and PBS. The phagocytic index was quantified by measuring the number of erythrocytes phagocytosed per 100 macrophages using bright field microscopy and 20x magnification, and inhibition is identified as percent of control. Data were collected from 4–7 independent experiments. The average control phagocytic index was 433.

To determine the phagocytic index for *Candida albicans* (Ca) and Cryptococcus neoformans (Cn) the macrophage like-cell line J774.16 (cultured in DMEM with 10% heat-inactivated FCS, 10% NCTC-109 medium, and 1% nonessential amino acids) was treated with 10 or 50 μM Methfor 2 h and then washed three times in media. As controls, J774.16 cells were incubated in medium alone or in the presence of 20 μM chloroquine. Ca strain SC5314 yeast cells were grown in brain heart infusion medium (BHI) at 37°C for 24 h then washed three times in PBS prior to application to macrophage. Cn yeast strain H99 was grown for 24 h in BHI at 37°C, incubated with capsule-specific monoclonal antibody (mAb) 18B7 [58] as an opsonin at 10 μg/ml for 2 h and then washed three times in PBS prior to incubation with macrophage. Ca and Cn cells were added to the macrophage monolayer at an effector to target ratio 1:1, and the suspension was incubated at 37°C for 30 min with Ca or 1 h with Cn. After incubation, remaining extracellular yeast cells were removed with three washes of PBS. The phagocytic index was determined by microscopic examination. For each experiment, five fields in each well were counted, and at least 100 macrophages were analyzed in each well. Wells were performed in triplicate for each condition examined.

Colony counts were made to determine the number of viable Ca and Cn yeast cells after phagocytosis. For the colony forming unit (CFU) determination, J774.16 macrophages were treated with or without Methor chloroquine and infected with Ca or Cn as described. The cultures were washed after 2 h to remove extracellular yeast and then incubated for an additional 22 h. After the 24 h total incubation, macrophage cells were lysed by forcibly pulling the culture through a 27-gauge needle 5 times. The lysates were serially diluted, and plated on Sabouraud dextrose agar at 37°C. CFU determinations were made after 72 h. Controls also consisted of yeast grown without macrophage, but in the presence of chloroquine or methamphetamine. All tests were preformed in triplicate.

## Supporting Information

Figure S1 Meth Does Not Affect Cell Viability Bone marrow-derived dendritic cells were treated with Meth (100 μM) or ethanol for 2 h at room temperature. Cells were then analyzed by flow cytometry for changes in cellular shape or granulosity both indicators of cell death. No differences in shape, morphology or granulosity were observed following Meth treatment.
(6.7 MB TIF)

Figure S2 Meth Does Not Affect Fluid Phase Phagocytosis Bone marrow-derived dendritic cells pre-treated with Meth or Clq for 2 h or left untreated were incubated for 30 min with PE-conjugated 2 μm fluorescent dextran microbeads with a cell-bead ratio of 1:100. The efficiency of the uptake was quantified by flow cytometry. Numbers in each quadrant refer to percentage of cells that have internalized 0, 1, 2, and 3 or more beads (from left to right).
(2.6 MB TIF)

Figure S3 Meth Induces Rapid Alkalization in Acidic Organelles in Macrophages (A) Representative fluorescence microscopy images of bone marrow-derived macrophages with reduced acridine orange staining following 10 min of Meth exposure Clq treatment. Scale bar, 10 μm. (B) Morphometric analysis of the decrease in acridine orange staining indicating alkalization upon 10 min treatment with Meth or Clq. Similar results were obtained in three independent experiments (*n* = 25–35, mean ± SEM, *p < 0.01, ***p < 0.0001, two-tailed ANOVA). (C) Progressive change in acridine orange label in the absence () or presence (▴) of 50 μM Meth. *p* < 0.001 for all time points versus control (*n* = 25–35, mean ± SEM, Student's two-tailed t-Test).
(5.6 MB TIF)

Figure S4 Meth Induces Autophagosome Accumulation in Macrophages as Lysosomal pH Is Alkalized (A) Progressive accumulation of GFP-LC3 positive autophagosomes in Meth- and Clq-treated cells at 2 h and 24 h. (*n* = 300–400, mean ± SEM, **p < 0.001, ***p < 0.0001, Fisher's exact test.) (B) GFP-LC3 labeled autophagosome formation in different concentrations of Meth after 24 h treatment. Similar results were obtained in three independent experiments. (*n* = 300–400, mean ± SEM, ***p < 0.0001, Fisher's exact test.) (C) Fluorescence micrographs of GFP-LC3 decorated autophagosomes in cells treated with Meth or Clq for 24 h. Arrow denotes examples of autophagosomes. Scale bar, 10 μm. (D) Phagosomes and autophagosomes labeled by GFP-LC3, in untreated, Meth- or Clq-treated (2 h) mouse peritoneal macrophages phagocytosing sheep erythrocytes. Red arrows denote representative autophagosomes, while yellow arrows denote representative phagocytosed erythrocytes. Scale bar, 10 μm. (E) Morphometric analysis of monodansylcadaverine label in peritoneal macrophages after 24 h treatment with Meth (*n* = 30, mean ± SEM, ***p < 0.0001, two-tailed ANOVA).
(4.8 MB TIF)

Figure S5 P24 Production Is Increased in Meth-Treated JR-CSF/huCycT1 Monocytes (A) Bone marrow dendritic cells from JR-CSF/huCycT1 mice were incubated with or without different concentration of Meth or NH_4_Cl. Supernatant was collected at day 7 and amount of secreted p24 measured. (Statistics *n* = 4 dishes, mean ± SEM, *p < 0.05, **p < 0.001, two-tailed ANOVA.) (B) JR-CSF/huCycT1 mice were left untreated or treated for one week with different amounts of Meth (low and high as described in materials and methods). Sera collected from each mouse were tested for amount of HIV-1 RNA using RT-PCR. The values are the mean and standard deviation from the values obtained from five mice in each group sample (*n* = 5 animals, mean ± SEM, two-tailed ANOVA).
(2.1 MB TIF)

Protocol S1 Supporting Methods (41 KB DOC)

Table S1 Fluid-Phase Phagocytotic Uptake of Lucifer Yellow (LY) Is Unaffected by Meth and Clq Treatment in Mouse Peritoneal Macrophages (1.6 MB TIF)

## Abbreviations

Ca: *Candida albicans*
Cn: *Cryptococcus neoformans*
Clq: chloroquine
[E(IgG)]: IgG-antibody coated erythrocytes
GFP-LC3: green fluorescent protein fused to microtubule-associated protein 1 light chain 3
LAMP-1: lysosome-associated membrane protein 1
Meth: methamphetamine
