# Correction: Methamphetamine Inhibits Antigen Processing, Presentation, and Phagocytosis

**DOI:** 10.1371/annotation/bd02ad26-a081-4c61-88c2-ebda285b8bca

**Published:** 2008-03-13

**Authors:** Zsolt Tallóczy, Jose Martinez, Danielle Joset, Yonaton Ray, Attila Gácser, Sima Toussi, Noboru Mizushima, Joshua D. Nosanchuk, Harris Goldstein, John Loike, David Sulzer, Laura Santambrogio

The eighth author's name should read: Joshua D. Nosanchuk. The first and second authors should not have been attributed equal contribution to the research.

