# Supplementary material for: Methamphetamine Inhibits Antigen Processing, Presentation, and Phagocytosis
Source: PLoS Pathog. 2008 Feb 15;4(2):e28. doi: 10.1371/journal.ppat.0040028 (PMC2242831; doi:10.1371/journal.ppat.0040028)
Supplement: Supplementary file 1 [file ppat.0040028.sd001.doc]

**Supporting Information**

**Supporting Methods**

**Meth cytotoxicity assay**

Dendritic cells (1x10^6^ cells) were incubated in the presence of 100 µM Meth or ethanol as control (since it was used to dilute Meth). After 2 hours cells were analyzed by flow cytometry. No differences in side scatter (SSC) or forward scatter (FSC) were observed between the two samples.

**Morphometric autophagy assay of monodansylcadaverine-labeled peritoneal macrophages**

Meth or Clq was added to the media, and the cells incubated for 2 h and 24 h before staining with monodansylcadaverine (Molecular Probes, Eugene, OR). monodansylcadaverine was added to the media at 100 µM final concentration. After 10 min incubation with monodansylcadaverine, coverslips were rinsed thoroughly with phenol red-free DMEM for fluorescence microscopy. Images were acquired using a Zeiss Axiovert 135 microscope, STAR I camera (Photometrics Ltd., Tucson AZ), IPLab Spectrum 3.1.1 image software (Signal Analytics, Vienna, VA), Zeiss Plan-NEOFLUAR 40x/1.3 Oil objective, Chroma 380 nm excitation filter and 525 nm emission filter set (Zeiss, Thornwood, NY). Morphometric analysis of 10-20 field images per coverslip was performed by measuring average pixel intensity in the cells using MetaMorph Version 6.1r6 image analysis software (Universal Imaging, Sunnyvale, CA).

**P24 production by Meth-treated JR-CSF/huCycT1 monocytes**

Monocytes were harvested from the bone marrow of JR-CSF/huCycT1 mice [44]. Mouse femurs were lavaged with phosphate-buffered saline (PBS). Cells were washed and resuspended in complete medium (RPMI medium containing 10% fetal calf serum). The cells were incubated in 10 cm tissue culture plates at 2 x 10^6^ cells/ml overnight at 37ºC. Recombinant mouse GM-CSF (20 ng/ml) was added. The next day, non-adherent cells were gently washed away and fresh media was added to the adherent monocytes on the original plates. Monocytes were then scraped, collected and spun down. 1 x 10^6^ monocytes in 2 ml of complete medium were added to each well in a 24 well plate. Three days later, cells were either left untreated or treated with different doses of methamphetamine (10, 50, 100, 150 µM) or NH_4_Cl (10 mM) every other day in quadruplicates. Recombinant mouse GM-CSF was added every 2 days. Supernatants were collected and analyzed for p24 antigen production by Elisa.

**HIV production by Meth-treated JR-CSF/huCycT1 mice**

JR-CSF/huCycT1 mice [44] were left untreated or treated intraperitoneally with increasing dosage of Meth over a 6 day period On days 0, 2, 4 and 6, each mouse received a 5 mg/kg dose. On days 8, 10 and 12, each animal received 6 mg/kg, 7mg/kg and 7.5mg/kg doses, respectively. Mice were sacrificed at the end of the treatment. HIV RNA was quantitated in 1:10 diluted sera from each mouse by RT-PCR (Roche Amplicor HIV-1 Monitor).

**Fluid phase pinocytosis**

Pinocytosis was measured using a Lucifer Yellow [57] assay in mouse resident peritoneal macrophages plated at a density of 3.5 x 10^5^ cells on glass cover slips. The cells were incubated in 300 μl PBS containing 0.5 mg/ml Lucifer Yellow, 0.1% Human Serum Albumin and 5.5 mM glucose for 2 h. Cover slips were then transferred to another 24-well tissue culture plate containing 100 μl distilled water, 0.05% Triton X-100 per well. Fluorescence was measured in a Cytofluor II fluorometer (485 nm excitation and 530 nm emission). Standard curves of Lucifer Yellow were generated from 0.05 to 0.003 mg/ml.
